# The Role of Gap Junctions in the Generation of Smooth Muscle Cells from Bone Marrow Mesenchymal Stem Cells

**DOI:** 10.1155/2022/1491327

**Published:** 2022-08-12

**Authors:** Ying Wang, Boping Yang, Pan Hu, Shentao Lu, Li Lei, Lubin Liu

**Affiliations:** Department of Obstetrics and Gynecology, Chongqing Health Center for Women and Children, Women and Children's Hospital of Chongqing Medical University, Chongqing, China

## Abstract

**Background:**

Studies have shown that stem cell transplantation can improve smooth muscle cell (SMC) regeneration and remodelling. Gap junctions can enhance the cytoprotective effects of neighbouring cells. We investigated the effect of gap junctions on the differentiation of bone marrow mesenchymal stem cells (BMSCs) into SMCs.

**Materials and Methods:**

Rat BMSCs and SMCs were obtained from the bone marrow and bladder of Sprague-Dawley rats, respectively. Flow cytometry and multilineage differentiation were performed to assess the characteristics of these cells. BMSCs and SMCs were incubated together in cocultures in the presence and absence of heptanol, an uncoupler of gap junctions. Cocultures were divided into three groups consisting of a contact coculture, noncontact coculture, and contact coculture plus heptanol groups. The expression of BMSC-specific markers and the effect of gap junctions on the differentiation of BMSCs were evaluated by performing real-time reverse transcription-polymerase chain reaction, immunofluorescence analysis, and western blotting after cocultures.

**Results:**

CD90 and CD44 were markedly expressed, and CD31 and CD45 were weakly or not expressed in BMSCs. The cells also showed good osteogenic and adipogenic differentiation ability. Compared with the noncontact coculture group, the SMC markers such as *α*-SMA, calponin, and connexin43 increased in the contact coculture group. The effect of contact in the coculture group was significantly weakened by heptanol.

**Conclusions:**

The results suggested that gap junctions play an important role in the generation of SMCs from BMSCs. The formation of SMCs can potentially be used to repair the sphincter muscle of patients with stress urinary incontinence.

## 1. Introduction

Over 200 million people throughout the world are affected with incontinence, a disorder that is linked to detrimental repercussions on society as well as a diminished quality of life [1]. One of the most prevalent types of incontinence, known as stress urinary incontinence (SUI), can be broken down into two primary subtypes: urethral hypermobility and intrinsic sphincter deficit. SUI is one of the most common types of incontinence recorded [2, 3]. Polytetrafluoroethylene, bovine collagen, silicone particles, carbon beads, and autologous ear chondrocytes have all been used in the short-term treatment of SUI, but the long-term results have yet to be determined [4]. However, these agents have the potential to bring about chronic inflammatory reactions, severe fibrosis, foreign body giant cell responses, periurethral abscess and particle migration, erosion of the urinary bladder and urethra, obstruction of the lower urinary tract with urinary retention, severe voiding dysfunction, and pulmonary embolism [5]. Smooth muscle cells (SMCs) are essential components of the sphincter muscle in the urinary system, and treatment with these cells can help patients with SUI [REFA]. In order to boost sphincter contractility, it is essential to design treatments that improve sphincter muscle activity. These procedures should be easily accessible, safe, cost-effective, and efficacious, and they should also be durable. A major limitation to such an approach is finding a reliable source of healthy SMCs that can be safely harvested and that would inflict minimal wounding.

Cells can be induced to undergo self-renewal and multipotent differentiation through the use of cellular injection therapy, which can ultimately lead to sphincter regeneration [6, 7]. Mesenchymal stem cells (MSCs) have been reported to differentiate into SMCs [8]. Additionally, it is possible for stem cells to secrete neurotrophins, which might then lead to the paracrine recruitment of endogenous host cells, which would simultaneously induce a regenerative response in nerve-integrated muscle [9, 10]. Sphincter function could be restored by pSMCs derived from human PSCs (hESC and iPSC) and may have paracrine effects. Bladder tissue engineering using MSCs shows better results than approaches using differentiated cells, and MSCs may migrate to bladder grafts and differentiate into SMCs [11]. Although bone marrow cells containing MSCs have been the most widely studied cell type in fundamental and clinical trials, the underlying mechanisms remain unclear, and the differentiation of MSCs into SMCs is not completely understood. The stem cell microenvironment or “niche” has been studied in order to explain stem cell lineage determination. Adult stem cells were observed to implant into a completely different germ layers and may differentiate into cell types similar to those of the injection environment [12]. Signaling molecules, the ability for cells to communicate with one another, and interactions between stem cells and the extracellular matrix that surrounds them are all components of a niche.

Gap junctions form channels between adjacent cells and provide a means to establish of direct intercellular communication [13]. As numerous physiological processes are mediated by regulatory molecules exchanged via gap junctions, the gap junction is considered as a key player that controls almost all aspects of the cellular life cycle [14]. Gap junctions have also been shown to enhance the cytoprotective effects of cardiomyocytes [15]. In this study, we investigated the role of gap junctions on the differentiation of bone marrow-derived MSCs (BMSCs) into SMCs. BMSCs can potentially be used to repair the sphincter muscle in the urinary system of patients with SUI.

## 2. Methods and Materials

### 2.1. Animals

Female 4-week-old Sprague-Dawley (SD) rats were obtained from the Third Military University Laboratorial Animal Center. All animals received humane care in compliance with the guidelines outlined by the Guide for the Care and Use of Laboratory Animals published by the US National Institute of Health (NIH Publication NO. 85-23, revised 1996). All experimental procedures were approved by the Care of Experimental Animals Committee of the Third Military Medical University.

### 2.2. Isolation and Culture of BMSCs and SMCs

Isolation and primary cultures of BMSCs from SD rats were performed as described by Sun et al. [16]. Briefly, rat BMSCs were isolated from bone marrow via density gradient centrifugation and were cultured in the Dulbecco's Modified Eagle Medium (DMEM)-F12 (Hyclone, China) supplemented with 10% fetal bovine serum, 100 U/mL penicillin (Gibco), and 100 *μ*g/mL streptomycin (Gibco) and incubated at 37°C in a 5% CO_2_ incubator. The first medium change was performed at 24 h. After that, the culture medium was removed and replaced with fresh medium every 3 days. After the cells had reached confluence, they were harvested by treating them with 0.25 percent trypsin (Hyclone), and then, they were passed on. All of the studies were carried out with cells that were between passages 2 and 4.

Rat bladder SMCs were isolated by using type II collagenase (Sigma, St. Louis, MO, USA) digestion as previously described [16]. The cells were cultured in high-glucose DMEM (Gibco), which was supplemented with 10% FCS, 100 U/mL penicillin, and 100 g/mL streptomycin. The cells were then incubated at 37 degrees Celsius in an incubator containing 5% carbon dioxide. The first medium change was performed at 48 h. After then, the culture medium was discarded and fresh medium was added at intervals of three days. Upon reaching confluence, the cells were harvested for passage with 0.25% trypsin. All experiments were performed using cells from passages 2-4.

### 2.3. Analysis of BMSC Characteristics

#### 2.3.1. Cell-Surface Antigen Determination

Passage 3 BMSCs were trypsinised with 0.25% trypsin and washed three times with phosphate-buffered saline (PBS) (Zhonshan Goldenbridge Biotechnology, Beijing, China). The supernatants were discarded, and the cells were suspended in 100 *μ*L of PBS. The cells were stained with monoclonal antibodies against fluorescein isothiocyanate- (FITC-) conjugated CD31, CD44, and CD90 (BD Biosciences, Franklin Lakes, NJ, USA) and phycoerythrin- (PE-) CY5-conjugated CD45 (BD Biosciences) according to the manufacturer's instructions. All incubations were performed at 37°C for 20 min. Controls were incubated with mouse IgG2a-FITC (JingMei Biotech, China) and mouse IgG1-PE-CY5 (JingMei Biotech) isotype antibodies, respectively. After incubation, the cells were washed with PBS containing 0.1% bovine serum albumin. Quantitative analyses were then performed using a Beckman Coulter flow cytometer (Brea, CA, USA).

#### 2.3.2. Multilineage Differentiation

BMSCs from passage 3 were analysed for osteogenic and adipogenic differentiation. Differentiation assays were performed as described previously [17]. Briefly, for adipogenic differentiation, BMSCs were grown to confluence for 3 weeks in DMEM-F12 containing 10% FCS, 1 *μ*M dexamethasone, 10 *μ*g/mL insulin (Invitrogen, Carlsbad, CA, USA), 200 *μ*M indomethacin, and 0.5 mM isobulyl-1-methylxanthione. The cells were fixed and stained with oil red O (Sigma-Aldrich) for 15 min. For osteogenic differentiation, BSMCs were grown to confluence 3 weeks in DMEM containing 10% FCS, 0.1 *μ*M dexamethasone, 10 mM *β*-glycerophosphate, and 50 *μ*M L-ascorbic acid-2-phosphate (Sigma-Aldrich). The cells were fixed and incubated in 1% alizarin red-Tris-HCl (Sigma-Aldrich) for 30 min to detect calcium deposits.

### 2.4. Culture Conditions

Cultures were performed in the following five experimental groups: SMCs without coculture (SMCs) group, a BMSCs without coculture (BMSCs) group, a direct contact culture (CON) group, a noncontact culture (NO-CON) group, and a direct contact with heptanol culture (CON+H) group. Experiments were conducted by using cell culture inserts (Millipore, Billerica, MA, USA) with a 1 *μ*m pore Biocoat PET membrane (surface area: 0.3 cm^2^). On coculture days 1, 2, and 3, total RNA was extracted from the upper BMSCs for performing real-time PCR. On coculture days 4 and 8, the total cell proteins from the upper chamber containing the BMSCs were collected for western blotting. On day 8, the membranes were carefully removed from the inserts for immunofluorescence analysis.

#### 2.4.1. SMCs without Coculture Group

The inserts were inverted onto the culture dish and subjected to rehydration in growth medium for 30 min, and then, SMCs in a cell suspension containing 2 × 10^5^ cells were applied to the upper membrane surface, and the mixture was then incubated at 37 degrees Celsius for six hours. The inserts were reverted and positioned into a 6-well culture plate (Corning, Inc., Corning, NY, USA), followed by incubation at 37°C in a humidified atmosphere of 5% CO_2_ until the SMCs reached confluence. These cells were the SMC group.

#### 2.4.2. BMSCs without Coculture Group

After rehydrating the inserts for 30 minutes in growth medium, a cell solution containing 2.5 × 10^5^ cells of BSMCs was applied to the upper membrane surface of the cell culture inserts. The inserts were then placed back into the growth medium. These cells were the BMSC group.

#### 2.4.3. CON Coculture Group

**Figure 1:**
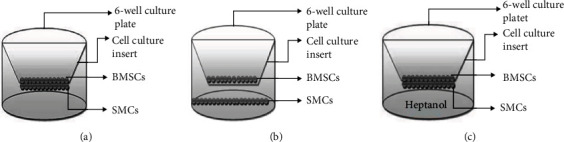
Diagrammatic representations of cell culture inserts containing the PET membranes. (a) Direct contact coculture group; (b) noncontact coculture group; (c) direct contact plus heptanol coculture group.

SMCs were grown to confluence on the inserts as described for the SMCs without the coculture group. A cell suspension containing 2.5 × 10^5^ cells of BSMCs was added to the upper membrane surface of the cell culture insert (Figure 1(a)). Growth media mixed 1 : 1 of the two cell types were used, and put the coculture system was incubated at 37°C in a humidified atmosphere of 5% CO_2_. The medium was changed every 2 days.

#### 2.4.4. NO-CON Coculture Group

SMCs were seeded into a 6-well culture plate and cultured in an incubator. When the SMCs reached confluence, the cell culture inserts that were previously rehydrated in growth medium for 30 min were placed in the 6-well culture plates. Then, a cell suspension of BSMCs consisting of 2.5 × 10^5^ cells was added to the upper membrane surface of the cell culture insert (Figures 1(b) and 1(c)). Growth medium was alternative by 5 : 5 mix of for the two cell types used. Finally, the coculture system was incubated at 37°C in a humidified atmosphere of 5% CO_2_, and the medium was changed every 2 days.

#### 2.4.5. CON+H Coculture Group

The CON+H group was the same as the CON group, except that 2.5 *μ*mol/L of the gap junction uncoupler heptanol (Sigma-Aldrich) was added to the growth media.

### 2.5. Immunofluorescence Analysis

The PET membranes were removed from the cell culture inserts on day 8 after the cells had been cultured. Following this, the cells were rinsed with PBS, fixed in paraformaldehyde at a concentration of 4% for 20 minutes at room temperature, and then washed once more with PBS. Nonspecific binding was blocked using 3% bovine serum albumin in PBS for 30 min. Then, membranes were incubated overnight at 4°C with a monoclonal mouse anti-human primary antibody to alpha smooth muscle actin (*α*-SMA) (1 : 100 dilution) (Santa Cruz Biotechnology, Dallas, TX, USA), calponin (1 : 200 dilution) (Abcam, Cambridge, UK), and connexin43 (Cx43) (1 : 200 dilution) (Abcam) in PBS. After washing with PBS, the membranes were incubated goat anti-mouse FITC-conjugated secondary antibody (1 : 100 dilution) (Beyotime Institute of Biotechnology, Shanghai, China) for 1 h at 37°C, and the nuclei were stained with DAPI (Beyotime Institute of Biotechnology). Thereafter, the membranes were washed, and the cover slips were mounted using Vectashield mounting medium. Samples were analysed by immunofluorescence microscopy using a confocal laser scanning microscope (Leica, Wetzlar, Germany).

### 2.6. Western Blot Analysis

After culturing the cells for 4 and 8 days, the underlying SMCs were scraped from the cell culture inserts and the inserts were washed with PBS. Protein extraction reagent (Pierce, Rockford, IL, USA) was added, and the BMSCs were transferred into microtubes. The supernatants were collected by performing centrifugation at 12,000 rpm for 20 min, and then, total protein concentrations were quantified by the Bradford assay. Equal amounts of protein were separated by performing SDS–polyacrylamide gel electrophoresis, and these were transferred onto polyvinylidene fluoride membranes. The membranes were incubated with monoclonal mouse anti-*α*-SMA (1 : 400 dilution), anti-calponin (1 : 500 dilution), and Cx43 (1 : 500 dilution) antibodies overnight at 4°C. The membranes were washed in PBS containing Tween 20 and then incubated with horseradish peroxidase-conjugated goat anti-mouse IgG (Zhonshan Goldenbridge Biotechnology) which was used as the secondary antibody, for 1 h at 37°C. After completion of the washing steps, the signals were developed using the ECL western blotting detection reagent. Proteins were visualised using Kodak X-OMAT film (Rochester, NY, USA), and band density was estimated by scan analysis using the ImageJ software.

### 2.7. Quantitative Real-Time PCR Analysis

After culturing the cells for 1, 2, and 3 days, the mRNA expression of *α*-SMA, calponin, and Cx43 in BMSCs of the different cocultured groups was quantified. Before performing extraction of the total RNA, the underlying SMCs were scraped from the cell culture inserts and the inserts were washed with PBS. Primer sequences in qPCR are as follows: *α*-SMA forward primer (5′⟶3′) TTCGTGACTACTGCTGAGCG and reverse primer (5′⟶3′) AGAAGAGGAAGCAGCAGTGG, valponin forward primer (5′⟶3′) AGTACCTCCCAGAACCGGAA and reverse primer (5′⟶3′) CATCCACAAACGCCAGTCAC, Cx43 forward primer (5′⟶3′) AAGCTCTGCGCTCCAAGTTA and reverse primer (5′⟶3′) CGCCAAAGTTGGTGGAACTC. Total cellular RNA of the BMSCs was extracted and reverse-transcribed using the TaqMan RT-PCR kit. Primer and probe sequences were designed by using GeneTools. First-strand cDNA was used as a template for conducting SYBR real-time PCR. Each PCR sample contained 12.5 *μ*L of the QuantiTect SYBR Green PCR Kit Master Mix (TOYOBO, Osaka, Japan) and 2.5 *μ*M primer pairs of each gene in a total reaction volume of 25 *μ*L. All amplifications were performed in triplicate and consisted of 45 cycles of 30 s at 94°C, 30 s at 60°C, and 45 s at 72°C using the ABI 7000 Prism Sequence detection system (Applied Biosystems, Foster City, CA, USA). The relative abundance of transcripts was normalised to the constitutive expression of GAPDH.

### 2.8. Statistical Analysis

All statistical analyses were performed using SPSS 15.0 for Windows (SPSS Inc., Chicago, IL, USA). The comparison of differences between groups was carried out using *t*-test analysis. A *P* value < 0.05 was considered to be statistically significant.

## 3. Results

### 3.1. Characteristics of Cultured BMSCs

**Figure 2:**
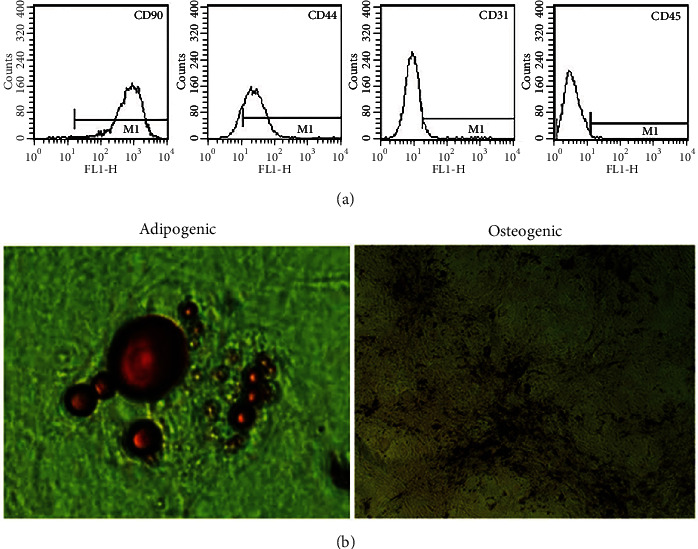
Flow cytometric analysis of cell-surface antigens of rat BMSCs at passage 3. (a) The positive rates of CD90, CD44, CD31, and CD45CD surface antigen expression in BMSCs were 99.57, 85.66, 6.0, and 0.35%, respectively. (b) Cells stained positive for oil red O and alizarin red and low-passage BMSCs showing adipogenic and osteogenic potential, respectively.

The cell-surface antigen profile of BMSCs from passage 3 was determined by staining with rat-specific monoclonal antibodies and flow cytometry. Expression levels of CD90, CD44, CD31, and CD45CD were 99.57, 85.66, 6.0, and 0.35%, respectively (Figure 2(a)). The high levels of CD90 and CD44 and low levels of CD31 and CD45 suggest that the BMSCs were undifferentiated stem cells, and that they differed from normal haematopoietic stem cells and endothelial cells. When the cells were evaluated for osteogenic and adipogenic differentiation after 3 weeks in under different conditions of culture. Osteogenic differentiation was reflected in the calcium deposition observed (Figure 2(b)), and the BMSCs formed adipose cells by differentiating adipogenic characteristics. These results demonstrated that the BMSCs can undergo multilineage differentiation.

### 3.2. Immunofluorescence of Gap Junctions Promoting the Differentiation of BMSCs into SMCs

**Figure 3:**
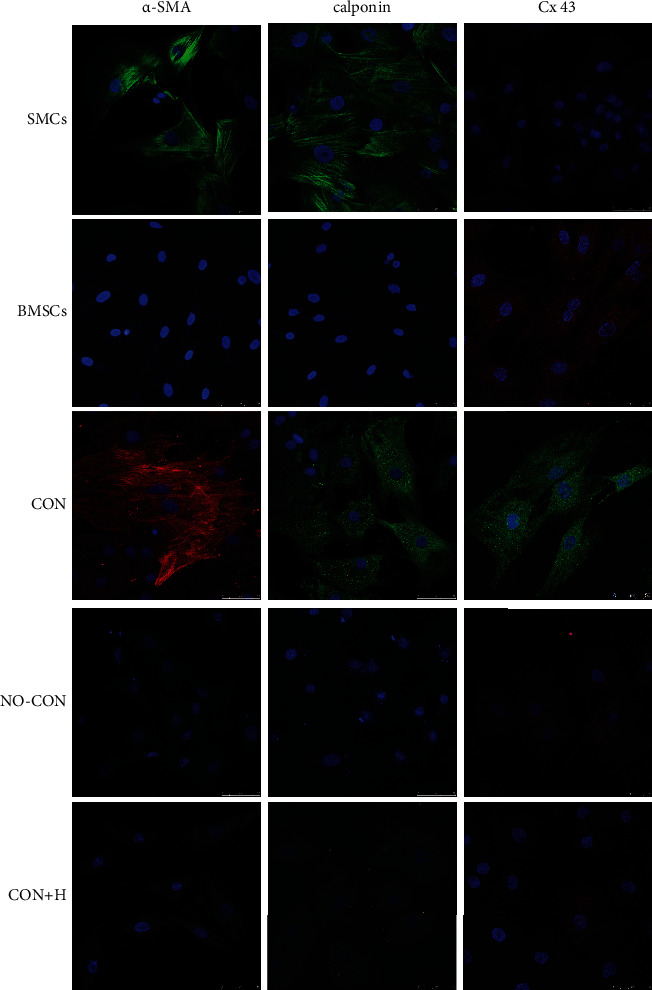
Expression of *α*-SMA, calponin, and connexin43 protein in BMSCs. Immunofluorescence photomicrographs after staining of cells with monoclonal antibodies for *α*-SMA, calponin, connexin 43 followed by goat anti-mouse FITC-conjugated secondary antibody after different coculture conditions (*n* = 3).

Immunofluorescence staining and laser confocal microscopy confirmed that markers *α*-SMA and calponin of SMCs were highly expressed on these cells, but the gap junction marker, Cx43, was minimally expressed. Pure BMSCs did not express *α*-SMA and calponin but weakly expressed Cx43. The BMSCs in the contact coculture group expressed *α*-SMA, calponin, and Cx43. The BMSCs in the noncontact coculture group did not express *α*-SMA and calponin and only expressed Cx43 weakly. Similarly, the BMSCs in the contact coculture group in the presence of the gap junction blocker, heptanol, did not express or only weakly expressed *α*-SMA, calponin, and Cx43 (Figure 3). These results suggest that the presence of gap junctions can promote the differentiation of BMSCs into SMCs.

### 3.3. Western Blot and Real-Time PCR Analysis of Gap Junctions Promoting the Differentiation of BMSCs into SMCs

**Figure 4:**
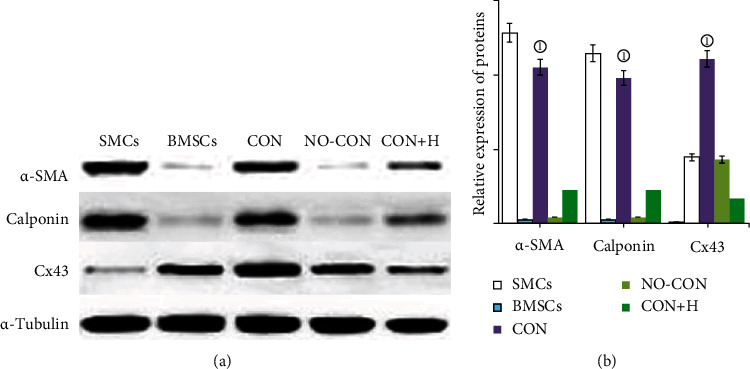
Effect of gap junction on the expression of *α*-SMA, calponin and connexin 43 protein in BMSCs by western blot analysis. (a) Western blot results after different coculture conditions (*n* = 3). (b) Quantitative analysis of the means and SEMs of 6 experiments (*n* = 3). ^①^*P* = 0.000, compared with the BMSCs, NO-CON, and CON+H groups.

**Figure 5:**
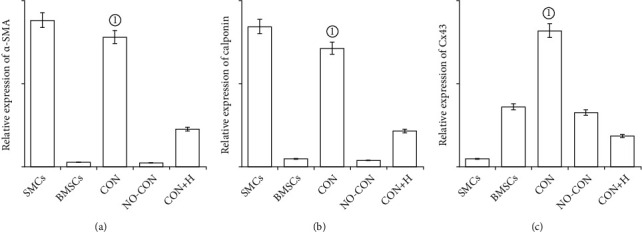
Effect of gap junction on the expression of (a) *α*-SMA, (b) calponin and (c) connexin43 mRNA in BMSCs by qRT-PCR analysis. Quantitative analysis of the means and SEMs of 6 experiments (*n* = 3). ^①^*P* = 0.000, compared with the BMSCs, NO-CON, and CON+H groups.

Western blot (Figures 4(a) and 4(b)) and real-time PCR (Figures 5(a)–5(c)) analyses showed that compared with the BMSCs and the noncontact and contact coculture groups with gap junction blocker added, the BMSCs in the contact coculture group were significantly different. There were significant differences in the expression of the SMC and gap junction marker genes and their encoded proteins, namely, *α*-SMA, calponin, and Cx43 (*P* < 0.001 in all cases). The results suggest that gap junctions between BMSCs and SMCs can promote the differentiation of BMSCs into SMCs.

## 4. Discussion

Cell transplantation therapy is a promising potential technique for regenerative repair of intrinsic sphincter deficiency which causes urinary incontinence [18, 19]. The use of autologous multipotent stem cells is the first preference in cell-based therapies and tissue engineering [20]. Stem cell therapy may replace, repair, or enhance the biological functions of damaged tissues or organs [21]. One commonly described source of SMCs is muscle-derived stem cells. Although these and satellite cells can be isolated by conducting biopsies in adults, the number of cells that can be harvested may be limited. BMSCs can be an ideal source of autologous adult stem cells, and these can be easily isolated and rapidly expanded using samples obtained from patients, without leading to major ethical and technical issues [22, 23]. Such cells have shown potential applicability as therapeutic agents. However, their application to intrinsic sphincter deficiency depends on the ability to control their differentiation into SMCs with high efficiency and purity.

BMSCs have the potential of self-renewal and multidirectional differentiation and can differentiate into cells required by their environment in different microenvironments [24]. The BMSCs obtained in this experiment obviously express the mesoderm phenotype markers CD44 and CD90 and low expression or no hematopoietic phenotype marker CD45 [25]. They have osteogenic and adipogenic differentiation characteristics, which are consistent with those reported in the literature. Studying the proliferation and differentiation potential of BMSCs into SMCs has important clinical significance for the treatment of various diseases including SUI [26]. The differentiation potential of BMSCs is regulated by multiple factors such as random inhibition or activation of transcription factors, microenvironment induction, and signal transduction pathways [27]. Among them, GJ plays an important role in regulating the osteogenic differentiation of BMSCs, but BMSCs differentiate into SMCs. The regulation of GJ needs to be further studied.

Gap junctions are tubular channels with an inner diameter of about 2 nm and are composed of 6 different types of connexins [28]. These proteins are the basic structural and functional proteins of gap junctions. Among them, Cx43 is widely distributed and abundant in mammals. It plays an important role in the transmission and material exchanges of BMSCs and helps in regulating the differentiation of BMSCs into osteogenic cells [29, 30]. However, its role in the differentiation of BMSCs into SMCs is still unclear. The structure of gap junctions allows the passage of cellular metabolites, signalling molecules (including PGE2, ATP, NAD, inositol derivatives, glutamic acid, and cyclic nucleotides) and ions to regulate cell proliferation and differentiation.

In this study, we developed a direct coculture system with a semipermeable membrane in order to investigate the effects of the local environment that are exerted on BMSC differentiation. We seeded SMCs under the membrane and BMSCs on the membrane and studied their interactions with respect to presence of gap junctions. The semipermeable membrane possesses 1 *μ*m diameter pores that do not allow cells to move across but can lead to the establishment of cell-cell contact and facilitate the diffusion of chemical factors. Using this system, we examined whether rat BMSCs could differentiate into SMCs after conducting direct cocultures using adult rat bladder-derived SMCs. We evaluated the expression of unique markers of SMCs, including *α*-SMA, calponin, and the gap junction protein, Cx43 [16]. We were able to detect the expression of *α*-SMA and calponin, which are early and middle markers of smooth muscle development, respectively, in cocultures of BMSC. The presence of these proteins was highly restricted to differentiated smooth muscle. We also found Cx43, which indicated that the gap junctions formed by the contact cocultures of BMSCs and SMCs can promote the differentiation of bMSCs into SMCs. These experiments confirmed that the BMSCs cultured in contact with SMCs highly expressed the marker genes of SMCs and their encoded proteins *α*-SMA, calponin, and Cx43.

## 5. Conclusion

The gap junctions formed by coculture of BMSCs and SMCs can promote the differentiation of BMSCs into SMCs. This initial study suggests that BMSCs can be used for cell transplantation therapy to generate SMCs. This is encouraging as BMSCs may potentially be used to repair the sphincter muscle in the urinary system of patients with SUI.

## Data Availability

The data used to support the findings of this study are available from the corresponding authors upon request.
